# Correction: Satisfaction with regular hospital foodservices and associated factors among adult patients in Wolaita zone, Ethiopia: A facility-based cross-sectional study

**DOI:** 10.1371/journal.pone.0273275

**Published:** 2022-08-15

**Authors:** Meskerem Teka, Gargi Dhar, Tadele Dana, Gedion Asnake, Negash Wakgari, Zeleke Bonger, Wakgari Binu Daga

The second author’s name is spelled incorrectly. The correct name is: Gargi Dhar. The correct citation is: Teka M, Dhar G, Dana T, Asnake G, Wakgari N, Bonger Z, et al. (2022) Satisfaction with regular hospital foodservices and associated factors among adult patients in Wolaita zone, Ethiopia: A facility-based cross-sectional study. PLoS ONE 17(3): e0264163. https://doi.org/10.1371/journal.pone.0264163
