# Supplementary material for: Satisfaction with regular hospital foodservices and associated factors among adult patients in Wolaita zone, Ethiopia: A facility-based cross-sectional study
Source: PLoS One. 2022 Mar 2;17(3):e0264163. doi: 10.1371/journal.pone.0264163 (PMC8890636; doi:10.1371/journal.pone.0264163)
Supplement: S1 Appendix — (PDF) [file pone.0264163.s001.pdf]

Supplementary file 1: Questionnaire in English, Amharic and Wolaita Donna. (PDF)

Questionnaire: English version

Interviewer: Please read the following paragraph to the respondent

Dear Respondent,

Good morning/good afternoon. Thank you for your willingness to with me today.

My name is------------------------------- I am working as a data collector in this study to assess patient satisfaction on regular hospital meal service and associated factors. Your name will not be written on this form, and the information that I got from you will never be used for other purposes. The data will keep confidential. Your frank answers will help us to better understanding patient satisfaction with Hospital meal service and to improve the provision of hospital meal service. We would greatly appreciate your participation in this study by genuinely responding to the interviews. The interview will take 20-30 minutes. Would you be willing to participate?

Agree_________________ Disagree_______________

Thank you.

Code number of questionnaire__________________

Name of interviewer__________________ Signature

Date of interview__________________

Name of supervisor __________________Signature__________________ Date_____________

Investigator: Meskerem Teka

Email address meskiteka@yahoo.com

Wolaita sodo university p.o. box 138

Part 1 Socio demographic			
101	Admission hospital	1. Ottona teaching Hospital<br>2. Dubbo hospital<br>3. Christian hospital	
102	How old are you	_______________ years	
103	Sex	1. Male<br>2. Female	
104	Where is your address?	1. Urban<br>2. Rural	
105	What is your Marital Status?	1. Single<br>2. Married<br>3. Divorced<br>4. Widowed	
107	What is your religion?	1. Orthodox<br>2. Protestant<br>3. Muslim<br>4. Catholic<br>5. Others specify	
108	What is your Occupation?	1. Government employee<br>2. Merchant<br>3. Student<br>4. Daily labor worker<br>5. Farmer<br>6. House wife<br>7. Others specify	
109	What is your Educational Status?	1. Unable to read and write<br>2. Can read and write but no formal education<br>3. Primary school completed<br>4. Secondary school completed<br>5. Higher education completed	
1010	How much is your monthly income in Ethiopian Birr /Average /	-------------------------in Birr	
1011	Number of days after admission	-------------------days	

**Part 2: Meal characteristic**			
		Yes	No
201	Do you get three meals daily?		

202	Is the meal flavoured enough to eat?		
203	Is there provision of water with your meal?		
204	Do you avoid meal because of meal taste aversion?		
205	Is the meal can be chewed easily?		
206	Do you skip meals because of new meal type?		
**Part 3: Serving Approach**			
301	Is the meal served at regular meal time?		
302	Are the meal served with clean dishes?		
303	Are the drinks served with clean Cups?		
304	Staffs who deliver your meals are neat?		
305	Staffs who deliver your meals are polite?		
**Part 4: Physical environment**			
401	Is the bed can be flexible in different position to eat?		
402	Is the room clean for you to eat in?		
403	Are there means of hygiene?		
404	Is the level of privacy in your room is arranged to eat freely?		

Part 5: Patient satisfaction: tick under the respective rate assigned as follows based on the patient response

1=Strongly disagree, 2=Disagree, 3= I don't know , 4=Agree, and 5=Strongly agree

	Satisfaction level	1	2	3	4	5
501	The meal served is testy					
502	The meal appearance is good					
503	The variety of the meal is enough					
504	The meal served is warm is good					
505	The time of meal distribution is timely					
506	The amount of the meal served is enough					
507	Dishes are clean					
508	Cups are clean					
509	Staff approach is good					
5010	Number of meals served per day is enough					
5011	The meal served is good for my health					
5012	The general cleanliness of the room is good					
5013	I am comfortable with the eating position					
5014	The level of room privacy is good					

Questionnaire: Amharic version

የስምምነት ማረጋጋጫ

ስሜ ---------------------- ይባላል፡፡ እኔ በሆስፒል ምግብና በምግብ አገልግሎት እርካታ ላይ በሚደረግ ጥናት አብሬ እሰራለዉ፡፡

የዚህ ጥናት ዓላማ በሆስፒታል ተኝተዉ ለሚታከሙ የሚሰጠዉን ምግብና የምግብ አገልግሎት በተመለከተ የህሙማንን እርካታ በማጥናትና አገልግሎቱ እንዲሻሻል ለማድረግ ነዉ፡ ፡በዚህም መሰረት እኔ አንዳንድ ጥያቄዎችን ለመጠየቅ እፈልጋለሁ፡፡ ስሞትን ሆነ ማንኛዉን ከእርሰዎ የምወስዳቸዉን መረጃዎች ለሌሎች ላለመስጠትና ለመጠበቅ ቃል እገባለሁ፡፡ ለጥያቄዉም ፈቃደኛ ባይሆኑም አናስገድዶትም ፡፡ በዚህም መብትዎ የተጠበቀ ነዉ፡፡ ለምናደርገዉ መጠይቅ ለሚያደርጉት ማንኛዉም ትብብር ከልብ እናመሰግናለን፡፡

የምንጠይቀዉን ቃለ መጠይቅ ለመመለስ ፈቃደኛ ነዎት ፣ አዎ ------------ አይደለም -----------

ፊርማ ------------------

የጥያቄዎች መለያ ቁጥር ------------------------

ቃለ መጠይቁን ያደረገዉ ሰዉ ስም --- ፊርማ ---------------------

ቃለ መጠይቁ የተደረገበት ቀን ---

የተቆጣጣሪዉ ስም ------------------------------- ፊርማ --------------------- ቀን---------------------

መመሪያ ፡- የሚሰጠዉ ምላሽ በፁሁፍ መሙላት ወይም ከቁጥሮች መርጦ በመክበብ ያሳዩ

መለያ ቁጥር -------------------------

ኢሜይል፡ meskiteka@yahoo.com

ወላታ ሶዶ ዩኒቨርሲቲ p.o. box 138

	መጠይቅ ክፍል 1 የቤተሰብ ማህበራዊ ገጽታ		
	ጥያቄ	በየክፍሉ የተሰጠ መልስ	**ምርመራ**
**101**	የተኙበት ሆስፒታል	**1.** ኦቶና ሆሰፒታል<br>**2.** ዱቦ ሆስፒታል<br>**3.** ክርስቲያን ሆሰፒታል	
**102**	ዕድሜዎት ስንት ነዉ ?	-----------------ዓመት	
**103**	ፆታ	**1.** **1.**ወንድ<br>**2.** **2.**ሴት	
**104**	አድራሻ	**1.** ከተማ<br>**2.** ገጠር	
**105**	የጋብቻ ሁኔታ	**1.** ያላገባ(ች)<br>**2.** ያገባ(ች)<br>**3.** የሞተችበት (ባት)<br>**4.** የፈታ(ች)	
**106**	ብሄረሰብዎ ምንድነዉ ?	**1.** ወላይታ<br>**2.** ጉራጌ<br>**3.** አማራ<br>**4.** ከንባታ<br>**5.** ሌሎች	
**107**	ሃይማኖትዎ ምንድነዉ ?	**1.** ኦርዶክስ<br>**2.** ፕሮቴስታንት<br>**3.** ሙስሊም<br>**4.** ካቶሊክ<br>**5.** ሌሎች	
**108**	ሥራዎ ምንድነዉ?	**1.** የመንግስት ሰራተኛ<br>**2.** ነጋዴ<br>**3.** ተማሪ<br>**4.** የቀን ሰራተኛ<br>**5.** ገበሬ<br>**6.** የቤት እመቤት<br>**7.** ሌላ ከሆነ ይግለጹ	
**109**	የትምህርት ደረጃዎት	**1.** ማንበብና መጻፍ የማትችል /የማይችል<br>**2.** ማንበብና መጻፍ የምትችል /የሚችል ግን መደበኛ ትምርት ያልወሰደች /ያልወሰደ<br>**3.** አንደኛ ደረጃ<br>**4.** ሁለተኛ ደረጃ<br>**5.** ከፍተኛ ደረጃ	

**1010**	በአማካይ የወር ገቢዎ ስንት ነዉ ፧(በዓይነት፤ በብር)	--------------ብር	

	**መጠይቅ ክፍል 2: የምግብ ባህሪያት**		
		አዎን	አይደለም
**201**	በቀን ሶስት ጊዜ ምግብ ያገኛሉ		
**202**	የምግቡ ጣዕም ጥሩ ነው		
**203**	ከምግብ አቅርቦት ጋር ውሃ ይቀርባል		
**204**	በምግብ ጣዕም ጥላቻ ምግብ ትተው ያውቃሉ		
**205**	ምግቦቹ በቀላሉ ይታኘካሉ		
**206**	ባለመዱት ምግብ ምክንያት ሳይበሉ ያልፉሉ		

	**መጠይቅ ክፍል 3: የምግብ አቀራረብ ሁኔታ**		
**301**	ምግብ በሰዓቱ ይቀርብሎታል		
**302**	ምግብ የሚቀርብበት ሳህን ንጹህ ናቸው		
**303**	መጠጫ እቃ ንጹህ ነው		
**304**	ምግብ የሚያቀርቡት ሰራተኞች ንጽህናቸውን ይጠብቃሉ		
**305**	ምግብ የሚያቀርቡት ሰራተኞች ባህሪ ትሁት ናቸው		

	**መጠይቅ ክፍል 4: የአካባቢ ሁኔታ**		
**401**	አልጋዎት የመንቀሳቀሱ ሁኔታ ምግብ ለመብላት ምቹ ነው		
**402**	የክፍሉ ንጽህና በውስጡ ለመመገብ አመቺ ነው		
**403**	ንጽህናዎን ለመጠበቅ በመታጠቢያ ክፍሉ የሚቀመጥ የንጽህና መጠበቂያ አለ		
**404**	እንደሚመቾት ለመብላት የክፍሉ ግላዊ ነጻነት ጥሩ ነው		

	**መጠይቅ ክፍል 5: ከምግብና የምግብ አገልግሎት ጋር የህሙማን እርካታን በተመለከተ**					
	**1.**በጣም አልስማማም **2.** አልስማማም **3.** እርግጠኛ አይደለሁም **4.** እስማማለሁ **5.** በጣም እስማማለሁ					
	የእርካታ ልኬት	**1**	**2**	**3**	**4**	**5**
**501**	በሆስፒታሉ የሚቀርቡልኝ ምግብ ጣዕም ጥሩ ነው					
**502**	የምግብ አቀራቡ ጥሩ ነው					
**503**	የምግቦች ዓይነት በቂ ነው					
**504**	የሚቀርቡት ምግቦች ትኩስ ናቸው					
**505**	ምግብ በሰዓቱ ይቀርባል					
**506**	የምግብ መጠኑ በቂ ነው					
**507**	የመመገቢያ ሰሃኖቹ ንጽህናቸውን የጠበቁ ናቸው					
**508**	የመመገቢያ መጠጫ ንጽህናቸውን የጠበቁ ናቸው					
**509**	የሰራተኞች ባህሪ ጥሩ ነው					
**5010**	በቀን የሚቀርበው የምግብ ጊዜ ብዛት በቂ ነው					
**5011**	የሚቀርበው ምግብ ለጤናዬ ጥሩ ነው					
**5012**	የክፍሉ ንጽህና ጥሩ ነው					
**5013**	የአልጋው ተንቀሳቃሽነት ምግብ ለመመገብ ይመቻል					
**5014**	የክፍል ግላዊ ነጻነት ጥሩ ነው					

Questionnaire: Wolaitegna version

pilggetta oosuwassi maaddiya oyshata

Maayettidoogaa maachchaa

Ta sunttay________________________geetettees. geetettiyariira Hosppitaaliya qumaanne qumaa haggaazan de`iya hanotaa issippe xanna`anawu maayaas. |xinaatiya oottanal.

Ha xinaatiyassi bayra halchchoy Hosppitaaliyan zin"idi aakimettiya hargganchchatussi imettiya qumaanne qumaa haggaazaa xeelliyagan harggiyagecttussi immiyo maaduwa xekkaa xanna`idi imettiya haggaazaa kaseegaappe loyttanaassa.

Hagaappe denddidaagan taani issi issi oysshata oychchana koyyayis. Intte sunttaakka gidin hara intteppe ekkiyo naqaashata ubbaakka hara asassi aattada immennaadan naaganawu geppaas. Oychchiyo oyshatussi zaaruwa immanawu eenana xayikkokka wolqqanttokko. Hagaabaggaara intte maatay bonchchettidaagaa. Nu shiishshiyo oyshaassi intte immiyo eenotaassi kechi wozanappe galatoos.

Nuuni oychchiyo oyshaa zaaranaayyoo eeno geetii?

Ee_______________Akkay|chii|______________paramaa_________________

Oyshatu kooddiy ________________

Oyshettiyaaoyshaaboxooxissidaagaa suntta______________________paramaa__________

Oyshay oyshettido gallassay___________________

Ha hanotaa kaallidi polissiyagaa sunttaa_________________paramaa______gallassaa_________

Kaalataa :Immiyo zaaruwa xuufiyan woykko paydotuppe dooridoogaa irzzoyidi bessite

Oyshaa kooddiya:________________________

Oyshaa shaahuwa 1: so Asaa Duussaa Hanotaa

M.P 1: He man”iyappe imettida			
	Oyshaa	He man”iyappe imettida zaarota	
101	Zin”idu Hosppitaalaa	Otona hosppitaaliya<br>Dubbo hosppitaaliya<br>kiristtiyaane hosppitaaliya	
102	Layttay woqqee?	_______________laytta	
103	mattumaa	1. Attuma___________<br>2. macca___________	
104	Qatuwa	1. 1.ambbaa<br>2. 2.ganddaa	
105	Ekuwa-geluwa hanotay	1. ekkibeenna/gelabaykku<br>2. ekkidaagaa/gelidaaro<br>3. hayqqidooga/hayqqidooro<br>4. birshshidaagaa/birshshettaaro	
107	Ammanoy/goynoy	1. Ortto-dokise<br>2. Pirotesttantte<br>3. Musilime<br>4. Katolike<br>5. Haray	
108	Ayba oosuulanaqeetii?	1. Kawo oosanchcha<br>2. Zal”anchcha<br>3. gallassa oosancha<br>4. goshshanchcha<br>5. keettaa ayssiyaro<br>6. hara ooso gidikko qonccissite	
109	Timirtte xekkay?	1. nabbabuwanne xaafuuwa danddayennaagaa<br>2. 1ro xekkaa<br>3. 2^{tto}xekkaa<br>4. 3^{tto}xekkaa	
1010	Aginaa damoozay woqqee?<br>biran/hara qommuwan	______________aginan	

**M.P 2: Qumawu de`iya eeshshaa(hanotaa)**		Ee	Akkay/chii/
201	qumaa issi galla heezzutoo?		
202	qumaassi peenoy 10”0?		
203	qumaara haattay issippe shiiqii?		
204	Imettiya qumaapeenoy injjetana xayin meennan aggido wodee de`ii?		
205	Imettiya qumati waayissennan coomettiyonaa?		
206	Meezetibeenna qumay shiiqido gaasuwan meennan aggido de`ii?		
**P.M 3: Qumaa shuuushshiyo hanotaa**			

		Ee	Akkay/chii/
301	Qumay saatiya naagidi shiiqii ?		
302	Qumay shiiqiyo miishshati geeshshee?		
303	Haatta uyiyo miishshati geeshshee?		
304	Qumaa shiishshiya oosanchchati bantta geeshshatetta naagiyonaa?		
305	Qumaa shiishshiya oosanchchatussi lo "o eeshshay de`ii?		
**M.P 4: Heeraa Hanta**			
401	Zin"ido hiixaa hanotay qumaa maanawu injjetii?		
402	Zin"ido kifiliya geeshshatettay qumaa maanawu injjetii?		
403	Udunxxee waayissido gaasuwan qumaa muussaa aggi ereetii?		
404	Qumaa bawu injjetiyatto maanawu he zin"ido kifiliyan buzo gidida la`atettay de`ii?		

**Oyshaa shaahuwa : Qumaanne qumaa haggaa zaara gayttidaagan aakimettiyageetussi de`iya lojje hanotaa xeelliyagan:**						
1=keehippe maayettikke, 2= maayettikke, 3=maacabeykke, 4=maayayis, 5= keehippe maayayis						
**P.M**	**Injje Hanotaa Likkiyo Mahata**	1	2	3	4	5
501	Hosppitaaliyan imettiya qumaa shonggay/peenoy/lo "o					
502	Qumaa shiishoy lo "o					
503	Qumaa qommati gidiya keena					
504	Shiiqiya qumati ho"iyageeta					
505	Qumay saatiya naagidi shiiqees					
506	Shiiqiya qumay kalissiyagaa					
507	Qumay shiiqiyo miishshati geeshshatettay naagettoogeeta					
508	Haattaa uyiyo miishshati geeshshatettay naagettoogeeta					
509	Oosanchchati lo"o eeshshay de`iyogeeta					
5010	Issi galla qumay shiiqiyo wodiya coratettay gidiyagaa					
5011	Shiiqiya qumay ta payyatettaassi lo"o					
5012	Zin"ido kifiliya geeshshatettay lo"o					
5013	Zin"ido hiixay qumaa maanawu injjetiyagaa					
5014	Zin"ido kifiliyassi de`iya buzo la`atettay lo"o					
