# Supplementary material for: Satisfaction with regular hospital foodservices and associated factors among adult patients in Wolaita zone, Ethiopia: A facility-based cross-sectional study
Source: PLoS One. 2022 Mar 2;17(3):e0264163. doi: 10.1371/journal.pone.0264163 (PMC8890636; doi:10.1371/journal.pone.0264163)
Supplement: Attachment — Submitted filename: Feedback_PONE-D-20-36652.docx [file pone.0264163.s002.docx]

**Dear Authors**

I would like to congratulate you for choosing an appropriate topic for research in healthcare setups. I have read complete manuscript. Please find below recommendations.

1) Add one separate section on **“Lessons for International Healthcare Managers”** at the end of manuscript
2) Mention type of hospitals (private or public) for 03 selected hospitals in abstract and other sections of manuscript.
3) Add one separate section on **“Overall Healthcare Facilities in Ethiopia”**, as a sub part of introduction section, which may include,
   a. number of private and public healthcare service providers in the country
   b. general comparison of quality of services between private and public service providers

**Regards,**

**(Reviewer)**
