# Supplementary material for: Satisfaction with regular hospital foodservices and associated factors among adult patients in Wolaita zone, Ethiopia: A facility-based cross-sectional study
Source: PLoS One. 2022 Mar 2;17(3):e0264163. doi: 10.1371/journal.pone.0264163 (PMC8890636; doi:10.1371/journal.pone.0264163)
Supplement: Attachment — Submitted filename: Response to Reviewers.docx [file pone.0264163.s003.docx]

**Manuscript ID:** PONE-D-20-36652

**Title**: Satisfaction with Regular Hospital Foodservices and Associated Factors among Adult Patients in Wolaita zone, Ethiopia: a facility-based cross-section study

**Journal**: PLOS ONE

**Dear Editor and Reviewers**,

Thank you for giving us an opportunity to revise our manuscript. We found your comments/feedback very helpful in improving the manuscript and we have revised the manuscript accordingly.

Here we tried to re-phrase our title a little bit by replacing ‘cross-section’ with “cross-sectional” as follows:

- Satisfaction with Regular Hospital Foodservices and Associated Factors among Adult Patients in Wolaita zone, Ethiopia: a facility-based cross-sectional study

Dear Dr. Daga,

Thank you for submitting your manuscript to PLOS ONE. After careful consideration, we feel that it has merit but does not fully meet PLOS ONE’s publication criteria as it currently stands. Therefore, we invite you to submit a revised version of the manuscript that addresses the points raised during the review process.

The manuscript has been evaluated by two reviewers, and their comments are available below. You will see the reviewers have congratulated you on the importance of your work. However, they have also raised a number of concerns that should be addressed before the manuscript can be further considered for publication.

The key concerns noted by the reviewers relate to the reporting of the study setting. Specifically, the reviewers requested additional information about the hospitals included in the study, as well as throughout Ethiopia, details about the food service, and clarity about the questionnaires and cut-off points.

Please submit your revised manuscript by May 29 2021 11:59PM. If you will need more time than this to complete your revisions, please reply to this message or contact the journal office at [plosone@plos.org](mailto:plosone@plos.org). When you're ready to submit your revision, log on to <https://www.editorialmanager.com/pone/> and select the 'Submissions Needing Revision' folder to locate your manuscript file.

Please include the following items when submitting your revised manuscript:

- A rebuttal letter that responds to each point raised by the academic editor and reviewer(s). You should upload this letter as a separate file labeled 'Response to Reviewers'.
- A marked-up copy of your manuscript that highlights changes made to the original version. You should upload this as a separate file labeled **'**Revised Manuscript with Track Changes'.
- An unmarked version of your revised paper without tracked changes. You should upload this as a separate file labeled 'Manuscript'.

If you would like to make changes to your financial disclosure, please include your updated statement in your cover letter. Guidelines for resubmitting your figure files are available below the reviewer comments at the end of this letter.

If applicable, we recommend that you deposit your laboratory protocols in [protocols.io](http://protocols.io/) to enhance the reproducibility of your results. Protocols.io assigns your protocol its own identifier (DOI) so that it can be cited independently in the future. For instructions see: <http://journals.plos.org/plosone/s/submission-guidelines#loc-laboratory-protocols>. Additionally, PLOS ONE offers an option for publishing peer-reviewed Lab Protocol articles, which describe protocols hosted on [protocols.io](http://protocols.io/). Read more information on sharing protocols at <https://plos.org/protocols?utm_medium=editorial-email&utm_source=authorletters&utm_campaign=protocols>.

We look forward to receiving your revised manuscript.

Kind regards,

Danielle Poole
Staff Editor
PLOS ONE

**Journal Requirements**:

When submitting your revision, we need you to address these additional requirements.

1. Please ensure that your manuscript meets PLOS ONE's style requirements, including those for file naming. The PLOS ONE style templates can be found at

<https://journals.plos.org/plosone/s/file?id=wjVg/PLOSOne_formatting_sample_main_body.pdf> and <https://journals.plos.org/plosone/s/file?id=ba62/PLOSOne_formatting_sample_title_authors_affiliations.pdf>

**Response**: Thank you for your guidance. We have revised the manuscript in accordance with the requirement.

2. Please include in your Methods section (or in Supplementary Information files) the participating hospitals/institutions.

**Response**: Thank you for your suggestion. We have included the participating hospitals in Methods section under the sub-title “Setting and Study Design”

3. We suggest you thoroughly copyedit your manuscript for language usage, spelling, and grammar. If you do not know anyone who can help you do this, you may wish to consider employing a professional scientific editing service.

 Whilst you may use any professional scientific editing service of your choice, PLOS has partnered with both *American Journal Experts (AJE)* and *Editage* to provide discounted services to PLOS authors. Both organizations have experience helping authors meet PLOS guidelines and can provide language editing, translation, manuscript formatting, and figure formatting to ensure your manuscript meets our submission guidelines. To take advantage of our partnership with AJE, visit the AJE website (<http://learn.aje.com/plos/>) for a 15% discount off AJE services. To take advantage of our partnership with Editage, visit the Editage website ([www.editage.com](http://www.editage.com/)) and enter referral code PLOSEDIT for a 15% discount off Editage services.  If the PLOS editorial team finds any language issues in text that either AJE or Editage has edited, the service provider will re-edit the text for free.

 Upon resubmission, please provide the following:

●The name of the colleague or the details of the professional service that edited your manuscript

●A copy of your manuscript showing your changes by either highlighting them or using track changes (uploaded as a *supporting information* file)

●A clean copy of the edited manuscript (uploaded as the new *manuscript* file)

**Response:** Thank you for your recommendation. However, it is difficult to use the recommended services, because the authors can’t afford the payment for the services. Instead, we have copy edited the whole manuscript for language usage, spelling and grammar with support from the communication experts at Wolaita Sodo University, consulting other researchers in the field, Wolaita Sodo University supported Office 365.

4. Please review your reference list to ensure that it is complete and correct. If you have cited papers that have been retracted, please include the rationale for doing so in the manuscript text, or remove these references and replace them with relevant current references. Any changes to the reference list should be mentioned in the rebuttal letter that accompanies your revised manuscript. If you need to cite a retracted article, indicate the article’s retracted status in the References list and also include a citation and full reference for the retraction notice.

**Response**: We acknowledged your comment. We removed the retracted references and re-phrased the paragraphs containing retracted references.

[Note: HTML markup is below. Please do not edit.]

**Reviewers' comments:**

Reviewer's Responses to Questions

Comments to the Author

1. Is the manuscript technically sounds, and do the data support the conclusions?

The manuscript must describe a technically sound piece of scientific research with data that supports the conclusions. Experiments must have been conducted rigorously, with appropriate controls, replication, and sample sizes. The conclusions must be drawn appropriately based on the data presented.

Reviewer #1: Yes

Reviewer #2: Yes

2. Has the statistical analysis been performed appropriately and rigorously?

Reviewer #1: Yes

Reviewer #2: Yes

3. Have the authors made all data underlying the findings in their manuscript fully available?

The [PLOS Data policy](http://www.plosone.org/static/policies.action#sharing) requires authors to make all data underlying the findings described in their manuscript fully available without restriction, with rare exception (please refer to the Data Availability Statement in the manuscript PDF file). The data should be provided as part of the manuscript or its supporting information, or deposited to a public repository. For example, in addition to summary statistics, the data points behind means, medians and variance measures should be available. If there are restrictions on publicly sharing data—e.g. participant privacy or use of data from a third party—those must be specified.

Reviewer #1: Yes

Reviewer #2: Yes

4. Is the manuscript presented in an intelligible fashion and written in standard English?

PLOS ONE does not copyedit accepted manuscripts, so the language in submitted articles must be clear, correct, and unambiguous. Any typographical or grammatical errors should be corrected at revision, so please note any specific errors here.

Reviewer #1: Yes

Reviewer #2: No

**Response**: We have corrected the typographical errors, the language usage, spelling and grammar with support from the communication experts at Wolaita Sodo University, consulting other researchers in the field, Wolaita Sodo University supported Office 365

5. Review Comments to the Author

Please use the space provided to explain your answers to the questions above. You may also include additional comments for the author, including concerns about dual publication, research ethics, or publication ethics. (Please upload your review as an attachment if it exceeds 20,000 characters)

**Reviewer #1**: Dear Authors

I would like to congratulate you for choosing an appropriate topic for research in healthcare setups. I have read complete manuscript. Please find below recommendations.

1) Add one separate section on “Lessons for International Healthcare Managers” at the end of manuscript.

**Response**: Thank you for your suggestion. We added the suggested section after the “Conclusion” section.

2) Mention type of hospitals (private or public) for 03 selected hospitals in abstract and other sections of manuscript.

**Response**: Thank you for your suggestion. We have mentioned the participating hospitals in “abstract and methods” sections.

3) Add one separate section on “**Overall Healthcare Facilities in Ethiopia**”, as a sub part of introduction section, which may include,
a. number of private and public healthcare service providers in the country
b. general comparison of quality of services between private and public service providers

**Response**: Thank you for your suggestion. We added the suggested section as part of our introduction. However, there were no officially published or clear report that may indicate the number of private healthcare facilities at national or local level in Ethiopia. Instead we discussed the available evidence on the specific number of public hospitals in Ethiopia.

Regards,
(Reviewer)

Reviewer #2: 1. Summary of the research

The study title is: “Satisfaction with Regular Hospital Foodservices and Associated Factors among Adult Patients in Wolaita zone, Ethiopia: a facility-based cross-section study”. The study was conducted in 3 hospitals and established that about a third (33.6%) of the patients were satisfied with hospital food services. Factors such as the patients’ residence, monthly income, flavor of the meal, and ease of chewing the food were associated with satisfaction with regular Hospital Foodservices.
This is a unique study, because there are limited studies of this nature in sub-Sahara Africa, and this has implications on the nutrition status of hospitalized patients, as well as the negative consequences of malnutrition in hospital, such as length of stay, mortality, and cost of treatment.

2. Examples and evidence

a. MAJOR ISSUES

• The authors can consider working with an editor to improve the quality of the manuscript. There are several grammatical and sentence structure issues throughout the document that should be addressed – a few examples include lines 50-51, 55-56, 88-89, 173-174, 180-181.

**Response**: Thank you for your comment. We have corrected the raised issues in the main manuscript.

Consider paying closer attention to Lines 181-187. The information conveyed is not clear due to the language, yet it is a key finding in the study that needs clear and concise communication. See highlighted area here …..“Accordingly, 417(98.6%) and 386(91.3%) of the patients reported that they were receiving three meals daily and chewable meals respectively. Study participants had unacceptable experiences on majority of the food characteristics they were asked. In relation to this, all (100%) of the participants reported that there was no provision of water with their meal, and 399 (94.3%) of patients say that the flavor of the meal was not enough to eat. In addition, significant portion of participants, 230(54.4%) and 155(36.6%) had avoided the meal because of taste aversion and had skip the meal because of new meal type respectively,……”

**Response**: We acknowledged the comment. We have corrected the raised issues by re-phrasing paragraphs in the main manuscript.

• Indicate the steps taken to ensure the validity of the data collection instruments as well as procedures. For example, what was the source of the questionnaires used?

**Response**: Thank you for your comment. We have revised to make clear the data collection related issues under the sub-section ‘Data collection and quality assurance’. It was tried to ensure validity of the tool as follows: “The tool for data collection was developed after reviewing literature (18-23, 33-35). The tool was developed in English then translated to local languages (Amharic and Wolaita Donna) and back translated to English with different experts. The tool was pre-tested on 5% of study participants at another hospital which was not selected for the actual data collection. In order to maintain the internal consistency of the tool and to determine the reliability of the test, Cronbach’s alpha was applied. If the alpha was high (≥0.70), the item is considered to be reliable and the test is internally consistent”.

• Indicate how the cut-off for the “overall patient (dis)satisfaction towards hospital foodservices” was determined (table 3). How was it decided that 142 were Satisfied, while 281 were Dissatisfied 281?

## Response: We have tried to make clear concerning the cut-off point for satisfaction level under the sub-sections “Measurements and Patient’s satisfaction level with regular hospital foodservices”.

• Consider rephrasing the lines 214-217, by using interpretive language for the readers to get the results in a clearer manner, as this is a key component of the study. For instance, rephrase the section on meal flavor, easily chewable food to something like, …. “those who reported that they liked the flavor of the food were 2.6 times more likely to be satisfied compared to those who reported that they did not like the flavor …”.

THIS IS DONE IN THE DISCUSSION SECTION. Consider moving some of the content from the discussion section to the findings, so that the discussion is focused on situating this study in existing literature, as well as the implication of the findings.

**Response:** Thank you for your guidance. We have corrected it in the manuscript.

• Consider merging contents of table 2 and table 3 that are presenting the same aspects of service. Currently, there is some overlap of information in the two tables. ONE SUGGESTION IS TO MAKE TABLE 2 AS CHARACTERISTICS OF SERVICE that were objectively determined e.g. number of meals, availability of water, availability of meal choice, whether they ate or skipped meals, among others. THEN IN TABLE 3, report all the subjective aspects that are the patients’ perceptions/ experiences.

**Response:** Thank you for your guidance. We have accepted the comment and corrected it in the manuscript. We have removed [Table 2] because the objectively determined services you suggested all are available in [Fig. 1]

• Consider giving a general description of the foodservices so that the reader has some context, e.g. cover things like whether there was a variety of foods to choose from?

## Response: We have described about the food variety of hospitals in a day in the main manuscript under sub-sections “Characteristics of foodservices in hospitals and Patient’s satisfaction level with regular hospital foodservices”.

• Consider doing analysis by hospital as a factor since the different hospitals may have different characteristics and experiences

**Response:** Thank you for your suggestion. We have tried to have admission hospital as factor but it had not made association in our binary logistic regression, the p-value was 0.359 while our plan was to have variables with p-value <0.25 as candidate variables for multivariable logistic regression analysis [see our plan under sub-section ‘Data analysis’].

b. MINOR ISSUES
• In the introduction, examples from similar settings, particularly African countries, can be included to allow for comparison for levels of dissatisfaction (lines 58 and 67).

**Response**: Thank you for your suggestion. We have included level of dissatisfaction among hospital clients in African countries

• The introduction can be boosted to include factors associated with (dis)satisfaction now that this is part of the title of the study

**Response**: Thank you for your suggestion. We have included level of dissatisfaction among hospital clients in African countries

• Provide/cite the references used for the population and sampling content

**Response**: Thank you for your suggestion. We have modified the content and included references.

• Lines 86-83 can be revised to describe the sampling process in a step-by-step clearer language.

**Response**: Thank you for your suggestion. We have made it clear by re-phrasing paragraphs.

• Give a better description of the hospitals that were included in the study

**Response**: Thank you for your comment. We have included descriptions of the selected hospitals for the study.

• Put ETB in full first before abbreviating, in lines 176-177

**Response**: Thank you for your comment. We have corrected accordingly.

• Table 1: Indicate the equivalence of Ethiopian Birr to USD for international readers

**Response**: Thank you for your comment. We have corrected accordingly.

• Line 199, the meaning of “means of hygiene (95.7%)” is unclear, consider rephrasing

**Response**: Thank you for your suggestion. We have re-phrased as “Hand washing facility”.

• Correct the spelling for “TASTE” from “TEST” in Figure 1 and in Table 3, row 2

**Response**: Thank you for your comment. We have corrected accordingly.

• If information on patients’ satisfaction with food texture and/ or temperature is available, this can be added to the findings

## Response: Thank you for your comment. There was information on the patient perception of meal temperature that asked patients to rate the warmness of the served meal under the sub-section “Patient’s satisfaction level with regular hospital foodservices”. However, there was no data concerning the food texture.

• The authors can ensure consistency in the way concepts are presented, by avoiding the interchanging of words that can result in different meanings in the text and tables. For instance, clarify whether when you are speaking with the patients on the chewability of the food (lines 182, 215, Table 4, Figure 1), was it that the food itself was tough to chew due to undercooking, or was it that the patients themselves had chewing issues, or both? The table 4 presentation versus that in the text (line 215) may differ in meaning

**Response**: Thank you for your suggestion. We have re-phrased as “well-cooked to chew easily”. Participants were asked to indicate whether the served meals were well-cooked to chew easily or tough due to undercooking.

• Clarify meaning of “means of hygiene” in line 193

**Response**: Thank you for your suggestion. We have re-phrased as “Hand washing facility”.

6. PLOS authors have the option to publish the peer review history of their article ([what does this mean?](https://journals.plos.org/plosone/s/editorial-and-peer-review-process#loc-peer-review-history)). If published, this will include your full peer review and any attached files.


If you choose “no”, your identity will remain anonymous but your review may still be made public.


**Do you want your identity to be public for this peer review?** For information about this choice, including consent withdrawal, please see our [Privacy Policy](https://www.plos.org/privacy-policy).

Reviewer #1: **Yes:** Dr. Vishal Kamra

Reviewer #2: **Yes:** Irene Ogada

[NOTE: If reviewer comments were submitted as an attachment file, they will be attached to this email and accessible via the submission site. Please log into your account, locate the manuscript record, and check for the action link "View Attachments". If this link does not appear, there are no attachment files.]

While revising your submission, please upload your figure files to the Preflight Analysis and Conversion Engine (PACE) digital diagnostic tool, <https://pacev2.apexcovantage.com/>. PACE helps ensure that figures meet PLOS requirements. To use PACE, you must first register as a user. Registration is free. Then, login and navigate to the UPLOAD tab, where you will find detailed instructions on how to use the tool. If you encounter any issues or have any questions when using PACE, please email PLOS at [figures@plos.org](mailto:figures@plos.org). Please note that Supporting Information files do not need this step.

*In compliance with data protection regulations, you may request that we remove your personal registration details at any time.* [*(Remove my information/details)*](https://www.editorialmanager.com/pone/login.asp?a=r)*. Please contact the publication office if you have any questions.*
