# Supplementary material for: Satisfaction with regular hospital foodservices and associated factors among adult patients in Wolaita zone, Ethiopia: A facility-based cross-sectional study
Source: PLoS One. 2022 Mar 2;17(3):e0264163. doi: 10.1371/journal.pone.0264163 (PMC8890636; doi:10.1371/journal.pone.0264163)
Supplement: Attachment — Submitted filename: Response for fig. 1 legends.docx [file pone.0264163.s004.docx]

PONE-D-20-36652R2
Satisfaction with Regular Hospital Foodservices and Associated Factors among Adult Patients in Wolaita zone, Ethiopia: a facility-based cross-sectional study
Mr. Wakgari Binu Daga

Dear Dr. Daga,

We've checked your submission and before we can proceed, we need you to address the following issues:

1. Please include a legend for figure 1.

**Response:** Thank you for your comment and we have addressed the raised issues in which we have put legend of the figure 1 at the end of the manuscript

We've returned your manuscript to your account. Please resolve these issues and resubmit your manuscript within 21 days. If you need more time, please email the journal office at plosone@plos.org. We are happy to grant extensions of up to one month past this due date. If we do not hear from you within 21 days, we will withdraw your manuscript.

Please log on to PLOS Editorial Manager at https://www.editorialmanager.com/pone/ to access your manuscript. You will find your manuscript in the 'Submissions Sent Back to Author' link under the New Submissions menu. Be sure to remove your previous manuscript file if you are uploading a new file in response to these requests. After you've made the changes requested above, please be sure to view and approve the revised PDF after rebuilding the PDF to complete the resubmission process.

We are requesting these changes to comply with the PLOS ONE submission guidelines (https://journals.plos.org/plosone/s/submission-guidelines). Please note that we won't send your manuscript for review until you have resolved the above requests.

Thank you for submitting your work to PLOS ONE and supporting our mission of Open Science.


Kind regards,

Audyssa Banlaygas Auditor
PLOS ONE

__________________________________________________
In compliance with data protection regulations, you may request that we remove your personal registration details at any time. (Use the following URL: https://www.editorialmanager.com/pone/login.asp?a=r). Please contact the publication office if you have any questions.
