# Supplementary material for: Satisfaction with regular hospital foodservices and associated factors among adult patients in Wolaita zone, Ethiopia: A facility-based cross-sectional study
Source: PLoS One. 2022 Mar 2;17(3):e0264163. doi: 10.1371/journal.pone.0264163 (PMC8890636; doi:10.1371/journal.pone.0264163)
Supplement: Attachment — Submitted filename: Feedback_PONE-D-20-36652_R2.docx [file pone.0264163.s005.docx]

**Dear Authors**

Congratulations !!!

All comments are addressed successfully.

**Best Wishes,**

**(Reviewer)**
